# Anatomical and Radiological Considerations When Colonic Perforation Leads to Subcutaneous Emphysema, Pneumothoraces, Pneumomediastinum, and Mediastinal Shift

**DOI:** 10.1055/s-0038-1624563

**Published:** 2018-02-22

**Authors:** Sala Abdalla, Rupinder Gill, Gibran Timothy Yusuf, Rosaria Scarpinata

**Affiliations:** 1 Departments of General Surgery and Radiology, King's College Hospital NHS Foundation Trust, London, United Kingdom

**Keywords:** colonoscopy, perforation, anatomical, radiology, pneumothorax, pneumomediastinum

## Abstract

While colonoscopy is generally regarded as a safe procedure, colonic perforation can occur and the risk of this is higher when interventional procedures are undertaken. The presentation may be acute or delayed depending on the extent of the perforation. Extracolonic gas following colonic perforation can migrate to several body compartments that are embryologically related and it has previously been reported in the thorax, mediastinum, neck, scrotum, and lower limbs. This review discusses in detail the anatomical pathways that led to a rare case of widespread subcutaneous emphysema, bilateral pneumothoraces, pneumomediastinum, and mediastinal shift from colonic perforation during a diagnostic colonoscopy. This is further supported by a description of the radiological images.

Lower gastrointestinal endoscopy is commonly used for the diagnosis, treatment, and monitoring of a range of benign and neoplastic colorectal diseases. While colonoscopy is the standard investigation for examining the whole colon, flexible sigmoidoscopy is an alternative for visualizing the distal third of the colon. In England, the rate of colonoscopy and flexible sigmoidoscopy procedures is 71.6 to 194.1 per 10, 000 population per year, with regional variation in the rest of the United Kingdom. 1

While colonoscopy is generally regarded as a safe procedure, complications such as bleeding and perforation and cardiorespiratory compromise can occur. The risk of colonic perforation is 0.2% in diagnostic colonoscopies and may be up to 10 times greater when therapeutic interventions such as polypectomies are undertaken. 2 3

Perforation during colonoscopy may occur through several mechanisms and extracolonic gas following colonoscopic perforation has previously been reported in the thorax, mediastinum, neck, scrotum, and lower limbs. 4 5 6 7 8 9 10 However, detailed discussions into the possible anatomical routes for the tracking of ectopic gas have not been undertaken.

This is a review of a rare clinical case of acute colonic perforation during colonoscopy that presented with cervical and truncal subcutaneous emphysema, bilateral pneumothoraces, pneumomediastinum, mediastinal shift, and cardiorespiratory arrest. A detailed discussion of anatomical planes and pathways that lead to this presentation is undertaken, which is further supported by a description of the radiological images.

## Clinical Case

An 83-year-old Caucasian female was scheduled for a colonoscopy under general anesthesia to investigate unexplained anemia (hemoglobin 115 g/L, mean cell volume 87.2 fL).

She had a background of hypertension, osteoarthritis, paroxysmal atrial fibrillation, and colonic diverticulosis. She had a past surgical history of total abdominal hysterectomy for uterine prolapse and right total hip replacement. She was independent in activities of daily living and had a performance status of 0.

To investigate the anemia, she was booked for oesophagogastroduodenoscopy (OGD), colonoscopy, and computed tomography (CT) scanning of the chest, abdomen, and pelvis to exclude malignancy.

The OGD was normal, and the CT scan revealed extensive diverticulosis in the sigmoid colon with associated narrowing and thickening of this segment of bowel. An outpatient colonoscopy was attempted under sedation but the patient was unable to tolerate the procedure. The procedure was abandoned and the patient was rescheduled for a colonoscopy under general anesthesia in the endoscopy suite.

On the day of colonoscopy, the patient was well. Induction of anesthesia performed by a consultant anesthetist was unremarkable. The colonoscopy was performed by a gastroenterology consultant and it was challenging because of poor bowel preparation and severe narrowing and tortuosity of a diverticular segment extending from the distal sigmoid to the mid descending colon. This segment was traversed, but at the level of the mid transverse colon, the patient's oxygen saturations dropped suddenly from 98% to 40% and there was an associated tachycardia of 160 beats per minute. Within 4 minutes of this sudden drop in oxygen saturation, the patient went into cardiac arrest, specifically pulseless electrical activity on the electrocardiogram monitor. The procedure was terminated and cardiopulmonary resuscitation (CPR) was commenced. The team that conducted the CPR noted that the patient had subcutaneous emphysema in the neck, chest and abdominal walls, and had a distended abdomen.

During the second minute of cardiac compressions, the patient was found to have reduced air entry and hyper-resonance on percussion of the right hemithorax. This was attributed to a right tension pneumothorax and this was decompressed with a wide-bore cannula placed in the second intercostal space in the right midclavicular line.

Upon decompression of the right tension pneumothorax, there was a return of spontaneous circulation and the oxygen saturation improved from 69% to 100%. An arterial blood gas revealed a pH of 7.36, partial pressure of oxygen of 15.2 on 15 L of oxygen, and a partial pressure of carbon dioxide of 6.08. A portable chest radiograph revealed bilateral pneumothoraces; the right-sided pneumothorax which was originally a tension pneumothorax had been converted to a simple pneumothorax, and on the left there was a simple pneumothorax. Two surgical chest drains were sited. The patient's temperature, pulse, and blood pressures were 36.0, 100 bpm, and 110/60 mm Hg, respectively. The abdomen once again was noted to be visibly distended and tympanic and there was a strong suspicion of colonic perforation.

Once stable, a CT scan of the chest, abdomen, and pelvis was obtained. This was reported by a radiology consultant as showing a large-volume pneumoperitoneum and free gas in the retroperitoneum with signs of perforation at the hepatic flexure of the colon. Note was made of bilateral pneumothoraces with extensive subcutaneous emphysema in the neck, chest, and abdominal wall and pneumomediastinum.

Shortly after this the patient was reviewed by the on-call surgical team and prompt arrangements were made for emergency laparotomy. At laparotomy, there was a perforation of the colon at the hepatic flexure with contained contamination around the proximal transverse colon and duodenum. A right hemicolectomy with exteriorisation of the ileal and colonic bowel ends as double-barrel stomas was carried out.

Postoperatively, the patient was managed on the intensive care unit (ICU) and required cardiovascular (ionotropic) and respiratory support by way of mechanical ventilation for the first 4 days. She was treated for a lower respiratory tract infection and required reintubation for a short duration. She also received hemodialysis for 5 days. She was eventually discharged from ICU on nutritional support after a 28-day stay. At this stage, her stoma was healthy and functioning, and there were no ongoing surgical concerns. She was managed on a respiratory ward and started making progress. However, 6 days after being moved to the ward she deteriorated acutely and rapidly from a lower respiratory tract infection. She died 2 days later, 35 days after her original colonoscopy.

## Discussion

Colonic perforation during colonoscopy may result from barotrauma, thermal injury, instrumental puncture of the bowel wall caused by the tip of the endoscope, or through interventions such as polypectomies and dilatation of strictures. The rate of perforation is variably reported and may be as low as 0.03% in diagnostic colonoscopies but up to 2.14% in therapeutic colonoscopies. 11 12 13 The sigmoid colon is the most susceptible site for perforation during endoscopy, since it may be exposed to excess shearing forces from the endoscope in addition to the fact that it is a common site for pathology such as polyposis, mass lesions, and diverticulosis. The cecum, with its thinner wall, is the second most common site for perforation. 13 14

In this case, the perforation occurred at the hepatic flexure of the colon, which is not a common location for iatrogenic perforation. The presence of pancolonic diverticulosis, poor bowel preparation, and the acute angle at the flexure may have been predisposing factors.

The risk factors for colonic perforation include increased age, female gender, multiple comorbidities, low body mass index, low plasma albumin level, underlying bowel pathology such as Crohn's disease and previous colonic resection, patients undergoing therapeutic procedures such as polypectomy, dilatation and endoscopic mucosal resection, and patients from an ICU setting. 2 11 15

Once colonic perforation has occurred, intraluminal gas may escape into the peritoneal cavity, the retroperitoneal space or both depending on the location of the perforation. Intraperitoneal perforations are the most common type. The ectopic gas may pass into different body compartments through distinct anatomical and fascial planes, as will be described below.

## Development of Pneumoperitoneum and Pneumoretroperitoneum

**Fig. 1:**
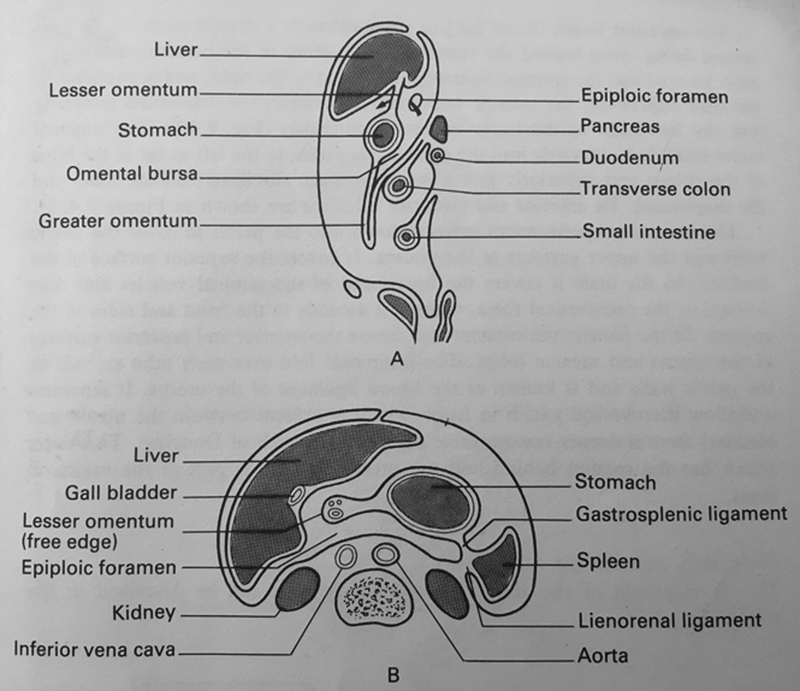
( **A** ) Sagittal section of the peritoneum and its reflections which form mesenteries and omental. The symphysis pubis, urinary bladder, and rectum are shown. ( **B** ) Peritoneum and its reflections in the axial section. (Adapted from Lumley et al 1987 14 ).

The peritoneum is a serous membrane which encases the peritoneal cavity. It consists of two layers: a parietal layer that covers the abdominopelvic wall and a visceral layer into which the viscera invaginate ( Fig. 1 ). The colon consists of four regions: ascending, transverse, descending, and sigmoid. The “retroperitoneal” ascending and descending parts are partially covered in peritoneum except posteriorly, where they are in direct contact with the posterior abdominal wall. The transverse and sigmoid portions are “intraperitoneal,” being completely covered by peritoneum and suspended by a double layer of this peritoneum, termed the transverse and sigmoid mesocolon, respectively. 16 17

**Fig. 2:**
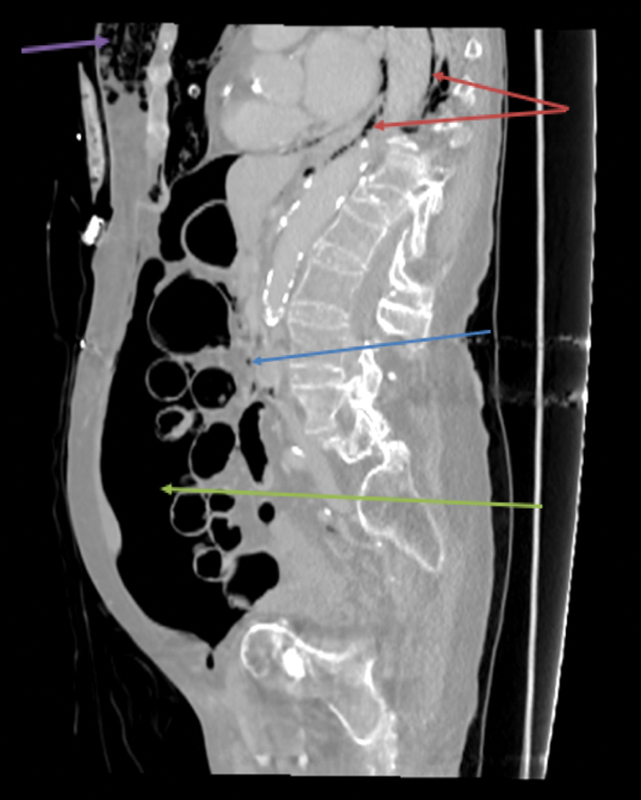
Sagittal computed tomography image demonstrating pneumoperitoneum (green arrow), air in retroperitoneum (blue arrow) and alongside aorta (red arrows) and subcutaneous emphysema in the anterior chest wall (purple arrow).

**Fig. 3:**
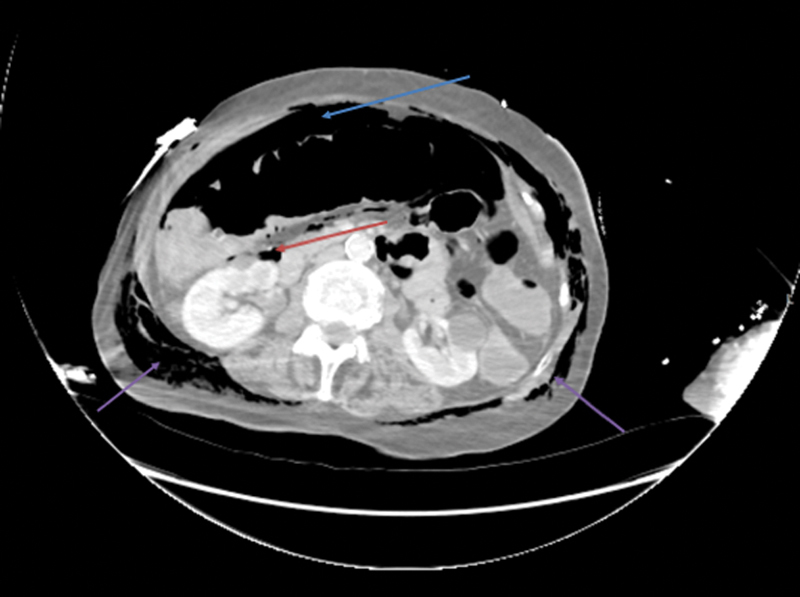
Coronal computed tomography image demonstrating free air in the peritoneum (blue arrow), retroperitoneum (red arrow), and subcutaneous emphysema in the abdominal wall around the flanks (purple arrows).

Anterior perforation at the hepatic flexure of the ascending colon, as in the case described above, led to gas escaping mainly into the peritoneal cavity ( Fig. 2 3 4 5 ). This was the result of a direct breach of the colonic bowel wall, which was confirmed on histology as an edematous opening in the mucosa. The CT scan demonstrated a large volume of free intraperitoneal and retroperitoneal gas ( Figs. 2 and 3 ). The retroperitoneal gas may have resulted from a concomitant posterior colonic wall perforation, leading to gas migration into the retroperitoneum, but the histological assessment of the specimen did not show evidence of this. It is, therefore, likely that the pneumoretroperitoneum resulted from the dissection of gas from the site of perforation through the colonic wall and subsequent passage along the mesocolon to the retroperitoneum.

**Fig. 4:**
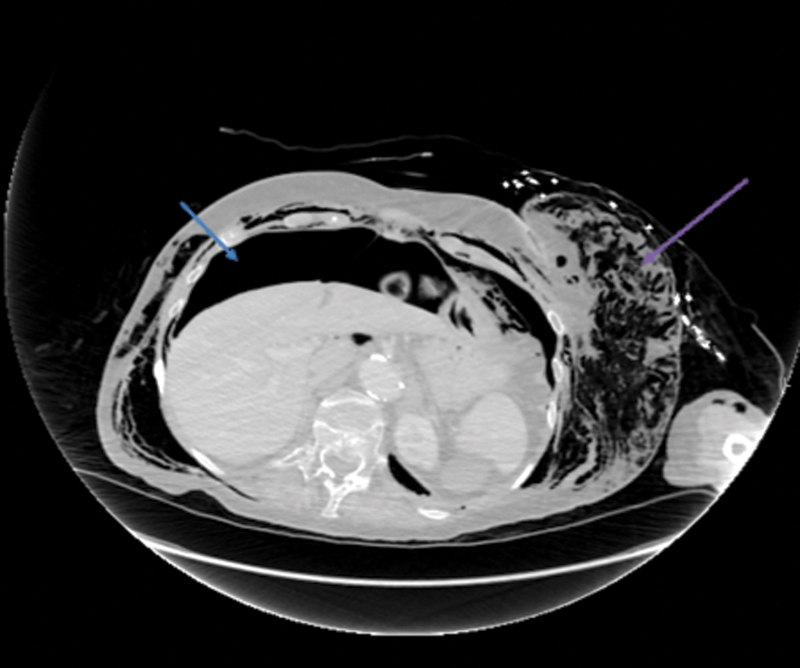
Axial computed tomography image demonstrating free gas in the peritoneal cavity (blue arrow) and subcutaneous emphysema in the left chest/abdominal wall (purple arrow).

**Fig. 5:**
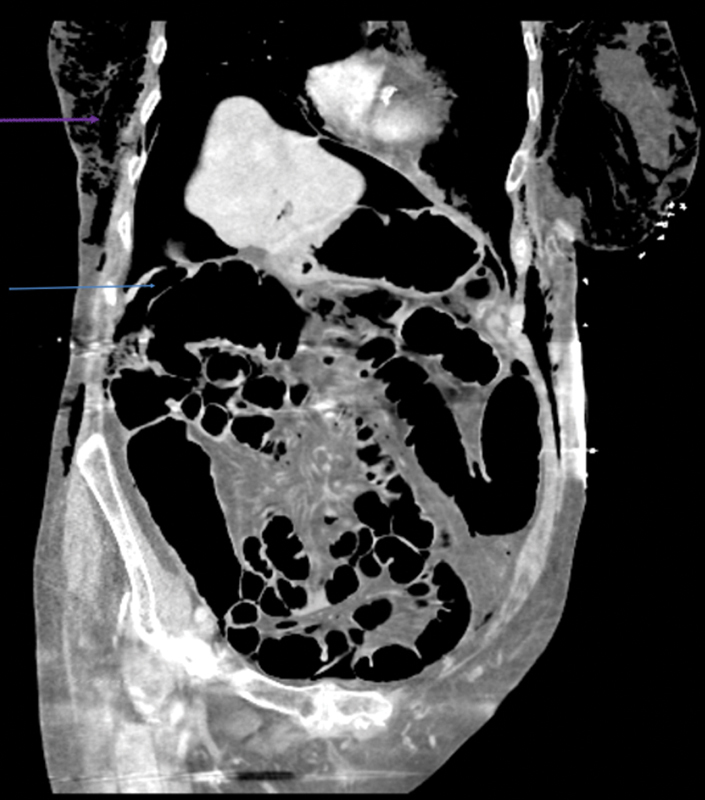
Coronal computed tomography image demonstrating free air in the peritoneal cavity (blue arrow) and subcutaneous emphysema in the thoracic and abdominal walls (purple arrow).

## Development of Pneumomediastinum

**Fig. 6:**
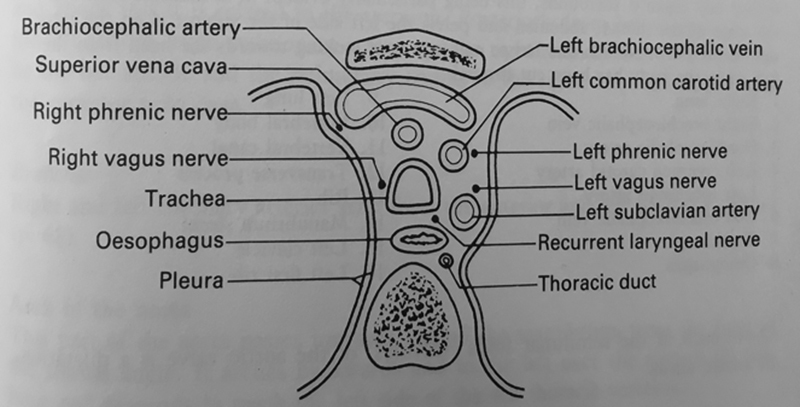
Structures seen on a transverse section through the mediastinum at the level of the 3rd thoracic vertebra (posterior) with the manubrium demonstrated anteriorly. (Adapted from Lumley et al 1987 14 ).

The mediastinum is the region in the thorax between the two pleural cavities ( Fig. 6 ). It contains the heart and great vessels, trachea, esophagus, thoracic duct, and the phrenic and vagus nerves. The mediastinum is generally subdivided into superior and inferior portions. The inferior portion is further subdivided into the anterior mediastinum, between the sternum and the heart; middle mediastinum, occupied by the heart and root of great vessels; and the posterior mediastinum, between the heart and the vertebral column, containing the trachea esophagus, descending aorta and the azygos vein. 16 17

The chest and abdomen originate from a single coelomic cavity during embryonic life. This cavity is lined by a serous membrane and the space deep to this membrane is the subserosal space. The coelomic cavity divides into the peritoneal, pleural, and pericardial regions. In the adult, the serous membrane of the peritoneum is the peritoneal membrane and the space below it is the subperitoneal or retroperitoneal space. The serous membrane of the thorax becomes the pleura, and the space deep to this is the subpleural or mediastinal space. The continuity of the subserosal space is maintained during subdivision of the coelomic cavity, forming the thoracoabdominal continuum which interconnects the subperitoneal (retroperitoneal) and subpleural (mediastinal) spaces. The mediastinal structures that traverse the diaphragm pass between these two spaces. Complete isolation of the thoracic and abdominal cavities occurs by means of the pleuroperitoneal folds by the 7th week of gestation. 18 19

**Fig. 7:**
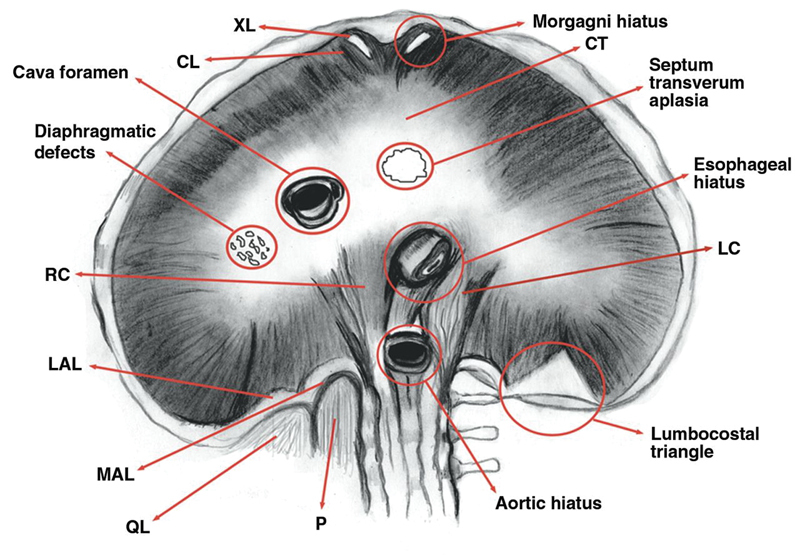
A view from the peritoneal (caudal) surface of the diaphragm demonstrating the various transphrenic pathways. The aortic, esophageal, and caval hiatuses are circled. The esophageal and aortic hiatuses are between the right (RC) and left (LC) crura. The Morgagni hiatuses are on either side of the xiphoid (XL) process and costal (CL) attachments of the diaphragm. The lumbocostal triangle is posterolateral and is related to the lateral arcuate ligament (LAL). The septum transversum lacunar aplasia and diaphragmatic defects, porous diaphragm syndrome, are circled. Other communications can occur in different sites of central tendon (CT). MAL, medial arcuate ligament; P, psoas muscle; QL, quadratus lumborum muscle on right. (Reprinted with permission from Lidid et al 1999 11 ).

The esophageal diaphragmatic hiatus is in the muscular part of the diaphragm at vertebral level T10 and it admits the esophagus along with the anterior and posterior vagal trunks ( Fig. 7 ). It is connected to the bare area of the liver, which is also in communication with the gastrohepatic ligament and the retroperitoneum. This serves as a potential pathway of spread of air and various disease processes between the retroperitoneum and mediastinum. The diaphragmatic hiatus of the descending aorta is at vertebral level T12 which admits the aorta into the retroperitoneum ( Fig. 7 ). Communication, therefore, exists between the retroperitoneum and the mediastinum by way of the periaortic and periesophageal fascial planes. In the present case, pockets of air alongside the aorta and esophagus were seen on the CT images ( Fig. 2 ).

The inferior vena cava passes through the central tendon of the diaphragm at vertebral level T8. Its wall is adherent to the margin of the diaphragmatic foramen and therefore does not provide an avenue for direct spread.

There are additional congenital defects in the diaphragm that could facilitate the passage of gas to the mediastinum. Two defects exist in the retrosternal region between the sternal and costal attachments of the thoracic surface of the diaphragm forming foramina on either side through which the internal thoracic vessels pass. These are called the foramina of Morgagni, which were first described in 1761, and are lined by peritoneum. A tear in their peritoneal lining in the presence of pneumoperitoneum can permit the passage of gas cranially through the diaphragm ( Fig. 7 ). 18 19 20

Between the costal and lumbar parts of the diaphragm are triangular areas covered by pleura superiorly and peritoneum inferiorly known as the lumbocostal triangles. These are weak areas in the diaphragm. Defects in these areas may cause congenital diaphragmatic hernia (Bochdalek hernia) or act as a potential trans phrenic route for the migration of gas from the abdomen, if there is a breach in the peritoneum. 19 20 Another pathway for the travel of gas into the mediastinum is through small fenestrations in the tendinous part the diaphragm, which may be congenital or acquired ( Fig. 7 ).

Therefore, gas or disease processes that follow these diaphragmatic hiatuses and defects will pass between subperitoneal (retroperitoneal) and subpleural (mediastinal) spaces, providing a route between the retroperitoneum and the mediastinum.

While there was no clear evidence of congenital diaphragmatic defects on the CT images of this patient, it is important to be aware of these as potential routes for spread of disease processes between the abdomen and mediastinum.

The patient underwent CPR and required needle thoracostomy and chest drain insertions for decompression of the pneumothoraces. This can be considered as another potential cause for the development of pneumomediastinum as well as subcutaneous emphysema in the neck.

## Development of Bilateral Pneumothoraces and Mediastinal Shift

**Fig. 8:**
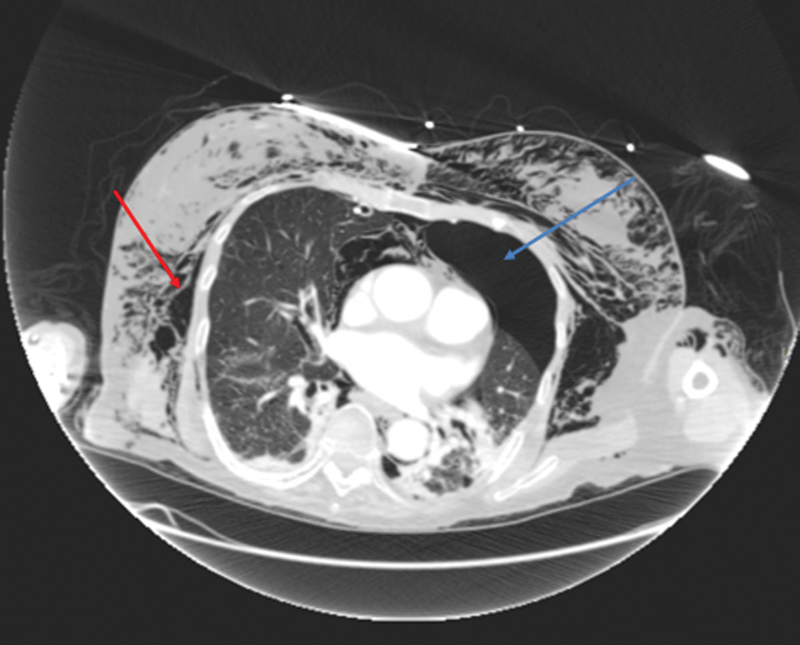
Axial computed tomography image of extensive subcutaneous emphysema in the thoracic wall (red arrow) and left pneumothorax (blue arrow).

**Fig. 9:**
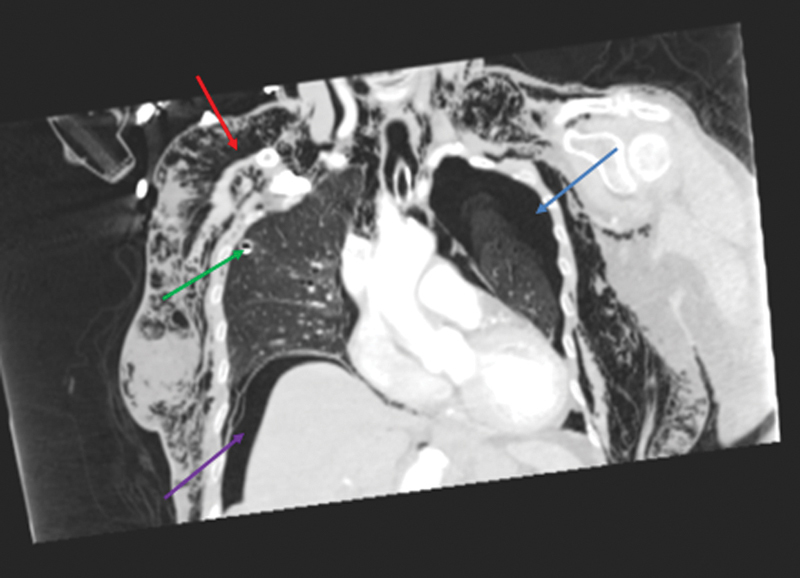
Coronal computed tomography image of extensive subcutaneous emphysema in the neck and thoracic wall (red arrow), left pneumothorax (blue arrow), and pneumoperitoneum (purple arrow). Part of the right chest drain is visible (green arrow).

Our patient developed a tension pneumothorax on the right and a simple pneumothorax on the left ( Figs. 8 and 9 ). Development of the tension pneumothorax is likely to have led to the sudden drop in blood oxygen saturation. A mediastinal shift would have resulted from the unilateral tension pneumothorax, and this is the most plausible explanation of development of the cardiorespiratory arrest.

The thoracic cavity contains the right and left pleural cavities and the mediastinum. The pleura is a double-layered serous membrane, and like the peritoneal membrane, it is divided into parietal and visceral layers ( Fig. 6 ). The parietal pleura lines the walls of the thoracic cavity, the lateral surface of the mediastinum, the upper surface of the diaphragm and extends superiorly above the thoracic inlet to cover the apices of the lungs. The visceral pleura covers the lungs and is continuous with the parietal layer around the root of the lung. 16 17

Several reports exist on the development of pneumothorax following colonic perforation. 4 5 7 9 10 21 22 These studies all describe the development of pneumothorax following colonoscopy through the initial movement of gas into the mediastinum from the peritoneum or retroperitoneum via continuous fascial planes, undiagnosed diaphragmatic defects, small fenestrations in the diaphragm or the aortic, and esophageal hiatuses. Once in the mediastinum, rupture of the mediastinal pleura causes decompression of gas into the pleural space, presenting as a pneumothorax. 4 8 9 10 13 If the flow of gas is rapid, a tension pneumothorax can develop. 21 22 However, evidence from the literature suggests that the reverse is not true in that a pneumothorax generally does not lead to pneumomediastinum. 8

Another point of consideration is that the pneumothoraces may have occurred from barotrauma as a result of intubation and ventilation. This may cause an air leak or rupture of subpleural bullae which may not necessarily present immediately after intubation and commencement of mechanical ventilation.

Direct communication between the pleural spaces and the peritoneum can develop in the presence of malformation or defects in the pleuroperitoneal membranes. 19 20 Although there was no evidence of this on the CT images of this patient, in such instances the air in the peritoneal cavity may pass through these defects directly into the pleural cavities. The presence of small defects in the tendinous part of the diaphragm is termed porous diaphragmatic syndrome and these act as pleuroperitoneal bullae. 10 18 Rupture of these bullae in the presence of a large volume pneumoperitoneum will allow gas to pass along a pressure gradient into the pleural cavity. Notable clinical examples of porous diaphragmatic syndrome are Meigs' syndrome (ovarian tumor, ascites and pleural effusion) and catamenial pneumothorax, where spontaneous pneumothorax occurs with the onset of menses 23 24

## Development of Subcutaneous Emphysema

The patient developed subcutaneous emphysema in the neck and the trunk as demonstrated on the CT images ( Figs. 2 3 4 5 , 8 9 10 ). There was palpable crepitus in the neck, chest, and abdominal walls. There are no barriers between the subcutaneous tissues of the body, which can act as a path of least resistance for gas migration. Following the colonic perforation, gas may have traveled along the mesentery to the abdominal wall and then spread to the chest and abdominal walls and subcutaneous tissues of the neck.

**Fig. 10:**
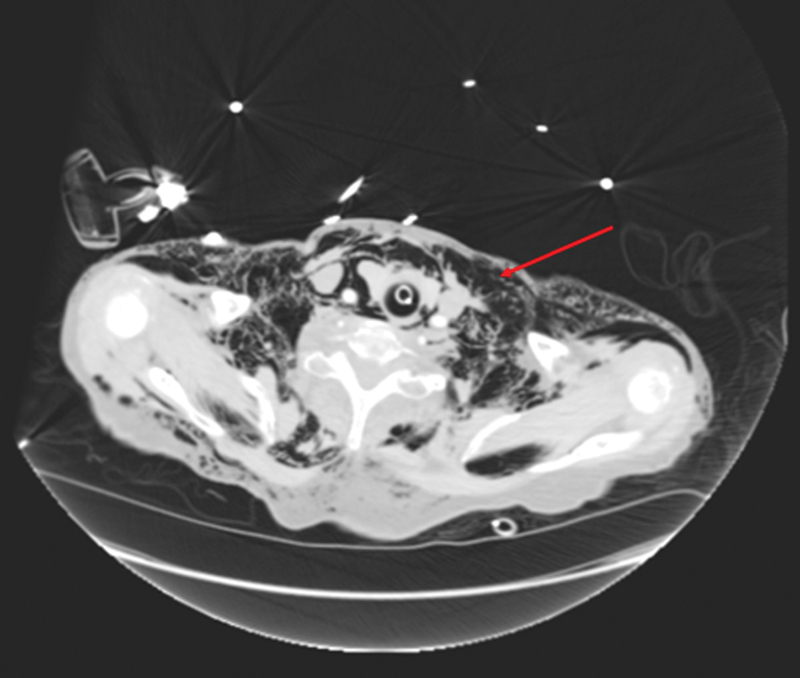
Axial computed tomography image of subcutaneous emphysema in the neck (red arrow).

## Conclusions

Anatomical continuity exists between the neck, thorax, and abdomen as has been demonstrated in this review of a rare case. This explains the pathogenesis of colonic perforation presenting with pneumothoraces, pneumomediastinum, mediastinal shift, and widespread subcutaneous emphysema. Recognition and prompt management of colonic perforation during colonoscopy save lives.
